# Telemedicine and Willingness to Die at Home in Rural and Remote Areas in Japan

**DOI:** 10.1177/26924366251382752

**Published:** 2025-09-24

**Authors:** Masanori Harada, Ryusuke Ae, Takao Kojo, Hiroya Masuda, Minoru Kibata, Hossain Mahbub, Tsuyoshi Tanabe

**Affiliations:** ^1^ Support Center for Rural Medicine, Yamaguchi Prefectural Grand Medical Center, Hofu, Japan.; ^2^ Division of Public Health, Center for Community Medicine, Jichi Medical University, Shimotsuke, Japan.; ^3^ Department of Health Sciences, Saitama Prefectural University, Koshigaya, Japan.; ^4^ Nagano Prefectural Shinshu Medical Center, Suzaka, Japan.; ^5^ Department of Public Health and Preventive Medicine, Yamaguchi University, Ube, Japan.

**Keywords:** telemedicine, end-of-life care, at-home death, rural and remote areas

## Abstract

**Background:**

Few studies have assessed the potential of telemedicine to improve end-of-life care quality in rural and remote areas. We investigated health care services needed for end-of-life care at home and examined their associations with willingness to die at home, testing the hypothesis that telemedicine facilitates at-home death in rural and remote settings.

**Methods:**

A cross-sectional survey was conducted with a target population of 6,382 residents aged ≥20 years living in designated rural and remote areas of Shunan City, Yamaguchi, Japan. Stratified random sampling was employed to select survey participants, resulting in 3,767 individuals who were mailed self-administered questionnaires. First, we assessed health care services needed for end-of-life care at home (telemedicine, home doctor visits, home nursing care, home personal care services, home support services, and senior day care). Multivariable logistic regression analysis was then performed to determine which services were independently associated with willingness to die at home.

**Results:**

Of 3,767 eligible participants, 1,884 (50.0%) responded, and 1,451 were included in the analysis. Among them, 608 (41.9%) were male, 1,166 (80.3%) were aged ≥60 years, and 733 (50.5%) expressed a wish to die at home. Home doctor visits were the most frequently selected service (776 participants [53.5%]), while telemedicine was selected by 193 (13.3%). After adjustment for all measured variables, telemedicine (adjusted odds ratio, 1.41 [95% confidence interval, 1.01–1.98], *p* = 0.045) and home doctor visits (adjusted odds ratio [OR], 1.50 [95% confidence interval, 1.19–1.90], *p* < 0.001) were independently associated with willingness to die at home.

**Discussion:**

These findings suggest that physician-provided services are central to enabling at-home death. Integrating telemedicine with traditional home doctor visits may improve end-of-life care and facilitate at-home death in rural and remote settings.

## Introduction

End-of-life care is a fundamental responsibility of health care providers in supporting dignified death in patients’ preferred places. Many patients wish to die at home rather than in hospitals because it allows for greater autonomy and closer family bonding.^1–5^ However, geographic disparities affect the quality of end-of-life care. Planning for at-home death in rural and remote medically underserved areas is particularly challenging because of limited availability and accessibility of health care resources.^6–10^

Previous studies have shown positive outcomes with telemedicine in rural and remote areas, including cardiovascular disease management,^11,12^ mental health services,^13,14^ emergency care,^15^ dementia care,^16^ palliative care,^17,18^ and patient satisfaction.^19–21^ In relation to at-home death, studies indicate that telemedicine-supported palliative care enables more patients to die at home as preferred, while also improving access to services and facilitating patient–provider communication.^22–24^ Although these studies^22–24^ did not specifically focus on rural populations, recent systematic reviews suggest that telemedicine may help improve the quality of end-of-life care in rural and remote settings.^25–28^

Building on these findings, we hypothesized that telemedicine facilitates end-of-life care and at-home death in rural and remote areas. To test this hypothesis, we first investigated which health care services (telemedicine, home doctor visits, home nursing care, home personal care services, home support services, or senior day care) may help enable end-of-life care at home in these populations. Second, we examined the association between telemedicine use and willingness to die at home.

## Materials and Methods

### Study setting, participants, and design

**FIG. 1.**
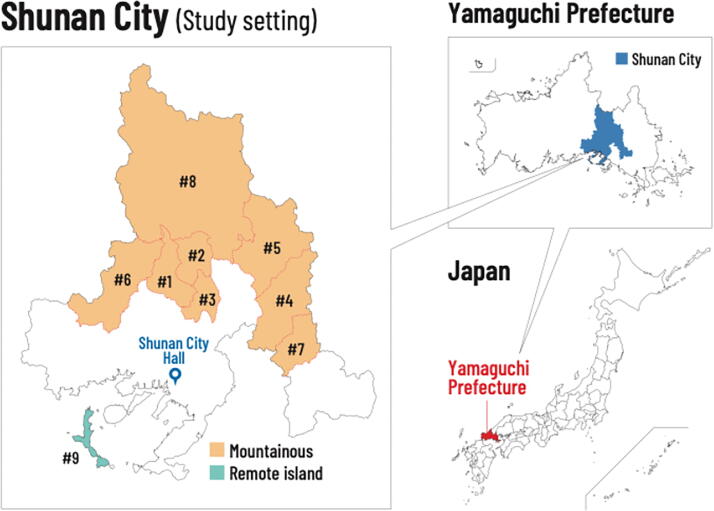
Study setting. Overview of Shunan City (2022): total population, 138,671; population aged ≥65 years, 68,168 (33%); habitable area, 656 km^2^.

We conducted a cross-sectional study using data from a questionnaire survey with a target population of 6,382 residents aged ≥20 years living in nine designated rural and remote areas in Shunan City, Yamaguchi Prefecture (Fig. 1). These areas included medically underserved areas, semi-underserved areas, and regions specifically designated by the city as having insufficient medical resources. Table 1 summarizes the geographical and demographic characteristics of these settings.

**Table 1.** Geographical and Demographic Characteristics of the Study Settings

Area	Population aged ≥20 years	Population aged ≥65 years (%)	Number of households	Habitable area, km^2^	Number of clinicians (employment type)	Medical care, days/week
#1	335	214 (61)	180	20	1 (Part-time)	1
#2	281	190 (65)	162	15	1 (Part-time)	1
#3	576	345 (56)	311	16	0	0
#4	576	363 (59)	339	37	1 (Part-time)	1
#5	258	193 (71)	167	49	1 (Part-time)	1
#6	1,067	608 (54)	592	42	1 (Part-time)	1
#7	558	318 (53)	299	19	1 (Part-time)	1
#8	2,535	1,484 (54)	1,520	181	1 (Full-time)	5
#9^a^	196	158 (80)	144	9	1 (Part-time)	2
Total	6,382	3,873 (57)	3,714	388	—	—

^a^ Area #9 is a remote island, while the others (#1–8) are mountainous rural and remote areas.

To minimize selection bias, we employed stratified random sampling for the selection of survey participants. First, based on the population size of each of the nine areas, we adjusted sampling rates as follows: 100% for seven areas with 350 or fewer residents, 45% for one area with 351–1,299 residents, and 20% for one area with more than 1,300 residents. Second, according to these rates, as well as sex and 5-year age-group distributions, we randomly selected individuals as survey participants. Finally, self-administered questionnaires were mailed to 3,767 individuals (59% of the target population), with responses collected from October 2022 through January 2023.

In Japan, the Basic Resident Registry is a comprehensive population registration system maintained by local governments that records all residents’ personal information including names, birthdates, sexes, and addresses. For this survey, participant identification and selection were conducted by Shunan City using the registry. Researchers only accessed fully anonymized participant data and did not have access to any personally identifiable information. This study was approved by the Tokai University Ethics Review Committee, and all participants provided informed consent (Approval ID: 22152, 7 October 2022).

### Variables

#### Background factors

We collected information on participants’ background factors as follows: sex (male or female), age (four groups: 20–39, 40–59, 60–79, and ≥80 years), residence area (two groups: remote island or other rural areas), household composition (three groups: living alone, living with spouse only, and living with family), mobile phone possession (three groups: no possession, feature phone, and smartphone), and current medical consultation status (two groups: regular outpatient visits or not receiving medical care).

#### Factors associated with end-of-life care

The survey questionnaire included variables related to end-of-life care, specifically “advance care planning (ACP)” and “wish to die at home.” ACP refers to the process of discussing future care preferences for patients who may lose decision-making capacity, and previous studies have shown that ACP can enhance the quality of end-of-life care.^29–32^ All participants were asked whether they had previously engaged in ACP discussions (yes or no). Participants were also asked whether they were willing to die at home, defined as “wish to die at home (yes or no),” which was the main outcome measure of this study.

#### Health care services needed for end-of-life care at home

We also gathered information on health care services considered necessary for end-of-life care at home, using the following question: “Which services are necessary to enable end-of-life care at home?” Six specific options were provided (telemedicine, home doctor visits, home nursing care, home personal care services, home support services, and senior day care), and multiple answers were allowed.

The survey questionnaire was newly developed for this study through a collaborative process involving physicians, nurses, and local government officials. Survey items and wording were finalized after collecting feedback from multiple community residents. Since this study focused on residents’ preferences and did not employ validated measurement instruments, no formal pilot testing or validation was conducted. A copy of the survey questionnaire (in Japanese) is available as Supplementary Appendix.

### Statistical analysis

First, we described the distribution of background factors, factors associated with end-of-life care, and health care services needed for end-of-life care at home. Second, respondents were classified into two groups based on their answer to the question about willingness to die at home (yes or no), and differences in proportions between the two groups were examined using chi-square tests. Finally, multivariable logistic regression analysis was performed to identify which health care services were independently associated with willingness to die at home, adjusting for all measured variables. In this regression model, the dependent variable was “wish to die at home,” and the independent variables included all six health care services as well as all other measured factors. Adjusted ORs with 95% confidence intervals (CIs) were calculated for all variables, and the results for health care services were visualized using forest plots. All analyses were performed using IBM SPSS Statistics for Windows, version 25 (IBM Corp., Armonk, NY, USA). The significance threshold was set at *p* < 0.05.

## Results

Of the 3,767 eligible survey subjects (i.e., participants who were mailed a questionnaire), 1,884 (50.0%) responded. After excluding those with incomplete responses for all measured variables, 1,451 participants (38.5% of all eligible subjects) were included in the analysis.

Table 2 presents the distribution of measured variables among study participants: 608 (41.9%) were male, 1,166 (80.3%) were aged ≥60 years, 71 (4.9%) lived on a remote island, 268 (18.5%) lived alone (without spouse or family), 893 (61.5%) owned a smartphone, 1,065 (73.4%) had regular outpatient visits, 651 (44.9%) had engaged in ACP discussions, and 733 (50.5%) expressed a wish to die at home. Regarding health care services considered necessary for end-of-life care at home, the most frequently selected was home doctor visits (776 participants [53.5%]), followed by home personal care services (774 [53.3%]), home support services (666 [45.9%]), and home nursing care (661 [45.6%]). Telemedicine was chosen by only 193 participants (13.3%).

**Table 2.** Variables of the Study (*N* = 1,451)

	*n* (%)
Background factors	
Sex	
Male	608 (41.9)
Female	843 (58.1)
Age, years	
20–39	71 (4.9)
40–59	214 (14.7)
60–79	800 (55.1)
≥80	366 (25.2)
Residence area	
Remote island	71 (4.9)
Other rural areas	1,380 (95.1)
Household composition	
Living alone	268 (18.5)
Living with spouse only	548 (37.8)
Living with family	597 (41.1)
No response	38 (2.6)
Mobile phone possession	
No possession	198 (13.6)
Feature phone	348 (24.0)
Smartphone	893 (61.5)
No response	12 (0.8)
Current medical consultation status	
Regular outpatient visits	1,065 (73.4)
Not receiving medical care	386 (26.6)
Factors associated with end-of-life care	
Advance care planning	
Experienced	651 (44.9)
Not experienced	745 (51.3)
No response	55 (3.8)
Wish to die at home	
Yes	733 (50.5)
No	718 (49.5)
Health care services needed for end-of-life care at home^a^	
Telemedicine	193 (13.3)
Home doctor visits	776 (53.5)
Home nursing care	661 (45.6)
Home personal care services	774 (53.3)
Home support services	666 (45.9)
Senior day care	584 (40.2)

^a^ Multiple responses were allowed.

When comparing factors between respondents with and without a willingness to die at home (Table 3), those who wished to die at home were more likely to be male (49.2% vs. 34.4%, *p* < 0.001) and aged 20–59 years (21.8% vs. 17.4%, *p* = 0.020). Participants living on a remote island and living alone were less likely to express a wish to die at home (remote island: 2.9% vs. 7.0%, *p* < 0.001; living alone: 15.5% vs. 22.6%, *p* < 0.001). In terms of health care services, telemedicine, home doctor visits, and home nursing care were more frequently identified as necessary for enabling end-of-life care at home among those who wished to die at home (telemedicine: 15.3% vs. 11.3%, *p* = 0.015; home doctor visits: 58.4% vs. 48.5%, *p* < 0.001; home nursing care: 48.4% vs. 42.6%, *p* = 0.015).

**Table 3.** Comparison of Variables by Willingness to Die at Home (*N* = 1,451)

	Wish to die at home		
	Yes [*n* = 733]	No [*n* = 718]	
	*n* (%)	*n* (%)	*p*-Value^a^
Health care services needed for end-of-life care at home			
Telemedicine			
Yes	112 (15.3)	81 (11.3)	0.015
No	621 (84.7)	637 (88.7)	
Home doctor visits			
Yes	428 (58.4)	348 (48.5)	<0.001
No	305 (41.6)	370 (51.5)	
Home nursing care			
Yes	355 (48.4)	306 (42.6)	0.015
No	378 (51.6)	412 (57.4)	
Home personal care services			
Yes	392 (53.5)	382 (53.2)	0.479
No	341 (46.5)	336 (46.8)	
Home support services			
Yes	326 (44.5)	340 (47.4)	0.147
No	407 (55.5)	378 (52.6)	
Senior day care			
Yes	288 (39.3)	296 (41.2)	0.243
No	445 (60.7)	422 (58.8)	
Other factors			
Sex			
Male	361 (49.2)	247 (34.4)	<0.001
Female	372 (50.8)	471 (65.6)	
Age, years			
20–59	160 (21.8)	125 (17.4)	0.020
≥60	573 (78.2)	593 (82.6)	
Residence area			
Remote island	21 (2.9)	50 (7.0)	<0.001
Other rural areas	712 (97.1)	668 (93.0)	
Household composition^b^			
Living alone	112 (15.5)	156 (22.6)	<0.001
Living with others	611 (84.5)	534 (77.4)	
Mobile phone possession^b^			
Smartphone	450 (61.7)	443 (62.4)	0.418
Others	279 (38.3)	267 (37.6)	
Current medical consultation status			
Regular outpatient visits	526 (71.8)	539 (75.1)	0.086
Not receiving medical care	207 (28.2)	179 (24.9)	
Advance care planning			
Experienced	323 (45.8)	328 (47.5)	0.269
Not experienced	383 (54.2)	362 (52.5)	

^a^ Chi-square test.

^b^ Participants with no response were excluded from the analysis.

**FIG. 2.**
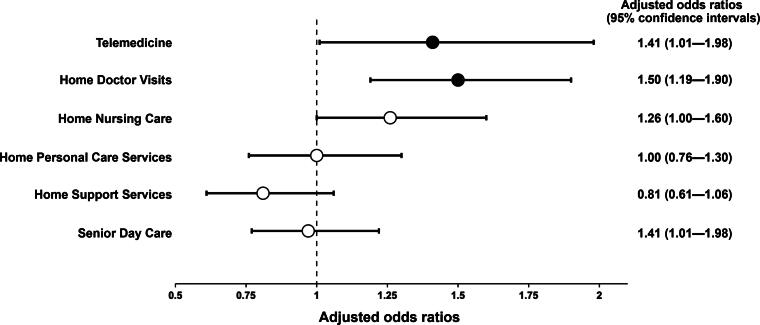
Association between health care services needed for end-of-life care at home and willingness to die at home (*N* = 1,451).

After adjustment for all measured variables (as potential confounders) (Fig. 2), multivariable logistic regression analysis demonstrated that telemedicine and home doctor visits were independently associated with willingness to die at home (telemedicine: adjusted OR 1.41 [95% CI, 1.01–1.98], *p* = 0.045; home doctor visits: 1.50 [1.19–1.90], *p* < 0.001). Adjusted ORs with 95% CIs for other covariates included in the multivariable logistic regression model are provided in Supplementary Table S1.

## Discussion

We conducted a self-administered survey among general residents living in rural and remote medically underserved areas in Japan and determined specific health care services needed for end-of-life care at home. We found that telemedicine and home doctor visits were independently associated with willingness to die at home. These findings support our hypothesis that telemedicine facilitates end-of-life care and at-home death in rural and remote settings. Importantly, our results indicate that rural residents perceive medical services *by physicians* as essential for at-home death, suggesting that integrating telemedicine with traditional home doctor visits could improve end-of-life care for residents who prefer to die at home in these areas.

Our study provides new insights into the role of telemedicine in end-of-life care among rural and remote populations. Haydon et al.^22^ reported that compared with people who had no video consultations, those who had at least one video appointment were more than twice as likely to die at home. Although their study was conducted in a metropolitan tertiary hospital,^22^ their findings align with our study. Furthermore, Breivik et al.^25^ found that telemedicine could support rural caregivers involved in family end-of-life care, and a review article by Mogan et al.^27^ suggested that telemedicine enhances the quality of end-of-life care in rural and remote communities. Our findings are consistent with and may extend these previous reports.^25,27^

Our results also showed that home doctor visits were strongly associated with willingness to die at home. This finding is supported by two studies focusing on end-of-life care at home. First, Tarasawa et al.^33^ analyzed the Japanese National Database and found that home doctor visits were significantly associated with higher proportions of death at home. Second, Krishnan et al.^34^ reported that home doctor visits were considered the most essential service among participants in a community-based primary palliative care program in India. Our results are consistent with these findings.^33,34^ Given that both telemedicine and home doctor visits were independently associated with willingness to die at home, combining these two services may be essential for enhancing preferred end-of-life care in rural and remote areas.

Home nursing care did not show a significant association with willingness to die at home in our multivariable analysis. However, previous studies have found that home nursing care services may enhance the quality of end-of-life care and facilitate death at home.^35,36^ Based on this evidence, our results may reflect a type II error (i.e., insufficient statistical power). Because our analysis showed marginal significance (*p* = 0.053) for the association between home nursing care and willingness to die at home, a larger sample size might have detected a true association. For the other health care services that showed no significant associations (home personal care services, home support services, and senior day care), it is possible that respondents could not clearly envision how these services would contribute to end-of-life care at home, as they do not directly involve medical professionals.

This study has limitations. First, the survey response rate was 50%. Nonrespondents may have influenced the results. Older adults who had difficulty completing questionnaires on their own may have been less likely to respond. Nevertheless, the use of stratified random sampling to minimize selection bias is a strength of this study, because few prior studies have applied such rigorous methods in rural and remote populations. Second, only a small proportion of participants had experience with telemedicine, which was not directly assessed in the survey. This lack of experience may partly explain the low percentage of participants (13.3%) who selected telemedicine as necessary for end-of-life care. Increased telemedicine use may alter these patterns, although previous research suggests that acceptance of telemedicine among older adults may be resistant to change.^37^ Third, this study was conducted in a single Japanese city. Generalizability to other rural and remote areas requires further investigation. Fourth, the questionnaire included only six specific health care service options, which may have introduced information bias. Finally, we did not assess whether any participants were currently in end-of-life situations. This study therefore assessed hypothetical preferences rather than preferences of individuals currently experiencing end-of-life care.

## Conclusion

We investigated specific health care services that may enable rural and remote populations to support end-of-life care at home and examined their associations with willingness to die at home. Our results demonstrated that telemedicine and home doctor visits were independently associated with willingness to die at home. The combination of these physician-provided services may help facilitate preferred end-of-life care in rural and remote medically underserved settings.

## Authors’ Confirmations

Conception and design of study: M.H., R.A., and T.K. Acquisition of data: M.H. and T.K. Analysis and/or interpretation of data: M.H., R.A., H.M., and M.K. Drafting of the article: M.H., R.A., T.K., H.M., and M.K. Revision of the article critically for important intellectual content: M.H., R.A., H.M., and T.T. All authors have contributed significantly to the work, have approved the article, and agree with the submission.

## Abbreviations Used

ACP: Advance care planning
CI: Confidence intervals
OR: Odds ratio
