# Supplementary material for: Telemedicine and Willingness to Die at Home in Rural and Remote Areas in Japan
Source: Telemed Rep. 2025 Sep 24;6(1):309–16. doi: 10.1177/26924366251382752 (PMC12547402; doi:10.1177/26924366251382752)
Supplement: Supplementary Appendix [file 26924366251382752_supplementary_appendix.pdf]

[問15] 身の回りのことができなくなった場合のご希望を教えてください。〈一つだけ〉

□介護サービスなどを利用して、できるだけ自宅にいる
□ご家族のところへ転居して介護を受ける
□介護サービス付きの住宅に転居する
□介護施設に入る
□介護環境の良いところに移住する
□その他（ ）

[問16] あなたの介護や人生の最期について、相談したことがありますか。〈一つだけ〉

（1）介護が必要になった場合の希望（介護の場所や内容等）について
□相談したことがある（□夫婦間で □夫婦以外のご家族で）
□相談したことがない→その理由をお聞かせください
□相談したいが、相談できていない
□相談できる家族がいない（亡くなっている等）
□相談するつもりがない
□その他（ ）

（2）人生の最期を迎えるにあたってのご希望（療養の場所や医療内容等）について
□相談したことがある（□夫婦間で □夫婦以外のご家族で）
□相談したことがない→その理由をお聞かせください
□相談したいが、相談できていない
□相談できる家族がいない（亡くなっている等）
□相談するつもりがない
□その他（ ）

[問17] 介護や人生の最期を過ごす場所として、希望されるのはどこですか。〈一つだけ〉

介護を受ける場所	人生の最期を過ごす場所
□自宅	□自宅
□介護してくれる家族の家	□介護してくれる家族の家
□病院	□病院
□介護施設	□介護施設
□その他（ ）	□その他（ ）

[問18] どんなサービスがあれば、自宅で最期を迎えられると思いますか。〈複数可〉

□医師によるテレビ電話による診療
□医師による定期的な訪問診療
□看護師による定期的な訪問看護
□ヘルパーなどによる定期的な身体介護（食事、お風呂、トイレなどの介護）
□ヘルパーなどによる生活支援（掃除、洗濯、買い物など）
□デイサービス（自宅から日帰りで施設に通い、体操や食事・入浴等のサービスを利用）
□電話相談事業（医療について電話で相談できるサービス）
□緊急通報サービス
□その他（ ）

暮らしや医療に関するアンケート（周南市）

依頼文の内容をご理解いただき、調査の回答、公表に同意していただけますか？

□同意する　□同意しない

[問1] あなたのことについて、教えてください。

地域	□大道理 □大向 □長穂 □中須 □須金 □大津島 □和田 □八代 □鹿野
性別	□男 □女
年齢	□20代 □30代 □40代 □50代 □60代 □70代 □80代以降
家族構成	□一人暮らし □家族と同居（中学生以下がいる）<br>□夫または妻と2人暮らし □家族と同居（中学生以下がいない）
介護	あなた自身は要介護（要支援）認定を受けていますか □はい □いいえ<br>家族の介護をした経験がありますか □はい □いいえ<br>※身の回りの生活の世話も介護にいれて考えてください。

■生活について

[問2] 食料品はどのようにして、手に入れていますか。〈複数可〉

□近くのお店で購入 □定期的に来る生協等で購入 □通販を利用
□定期的に買い出し □ご近所に頼む □ご家族に頼む □自給自足（野菜・魚など）
□その他（ ）

[問3] 交通手段は何を利用していますか。〈複数可〉

□徒歩（杖・車いす・シルバーカー・歩行車を含む） □電動カート □自転車
□バイク □自家用車 □バス □タクシー □船 □その他（ ）

[問4] 携帯電話は、ご利用されていますか。〈複数可〉

□持っていない □携帯電話（スマホ以外）を利用 □スマートフォンを利用

[問5] 日常生活で、何かお困りのことはありますか。

[問6] 最寄りの市民センター・集会所などを利用する機会がありますか。

□ある □ない
※「ある」と答えた方は、どれくらいの頻度で利用しているか教えてください。
□月1日 □月2～3日 □週1～2日 □週3～4日 □週5日以上
□その他（ ）

■最寄りの診療所のご利用について

[問7] 市が運営する公設診療所に、内科以外で希望する診療科はありますか。〈複数可〉

□整形外科 □眼科 □耳鼻咽喉科 □皮膚科 □歯科 □産婦人科
□その他（ ）

中面もご覧ください

■診療所や病院のご利用やご希望について

[問8] 診療所や病院に、定期的に通院されていますか。〈一つだけ〉

□通院していない
□通院している
①通院方法を教えてください。
□徒歩　□電車・バス・船　□自転車　□自家用車　□家族に連れてきてもらう

②自宅から最も利用する診療所（病院）までの通院時間（片道）を教えてください。
□10分以内　□20分以内　□30分以内　□1時間以内　□1時間以上

③困った時に相談する医療機関名と受診頻度を教えてください。

医療機関名：＿＿＿＿＿＿＿＿　受診頻度：＿＿＿＿＿＿

④上記以外で通院している医療機関を教えてください。

	医療機関名（例：山口内科）	受診頻度（例：1週間に1回）
1		
2		
3		
4		

[問9] 医師以外で、病気について相談したい時は、誰に相談していますか。〈複数可〉

□看護師　□保健師　□ケアマネジャー　□地域包括支援センター　□家族　□友人
□インターネット　□ヘルパー　□民生委員　□その他（　　　　　　　　　　　）

[問10] 夜間や休日、休診日で、急な病気やけがの場合に連絡する方法が、119や家族・知人以外にありますか。〈一つだけ〉

□ない
□ある
※「ある」と答えた方は、知っている方法を教えてください。〈複数可〉
□支所に電話　□消防団に電話　□自治会長・民生委員に連絡
□その他（　　　　　　　　　　　）

[問11] 夜間や休日、休診日で、急な病気やけがの場合に医師や看護師に電話して相談できる方法があるのを知っていますか？

□知らない
□知っている
※「知っている」と答えた方は、知っている方法を教えてください。〈複数可〉
□休日夜間診療所に電話　□#7119に電話
□その他（　　　　　　　　　　　）

医師と会って診療を受ける方法（対面診療）に加えて、テレビ電話を使用した診療（オンライン診療）を追加する場合について、ご意見をお伺いします。

[問12] かかりつけの診療所や市民センター・集会所で、看護師が隣にいながら、テレビ電話で、かかりつけの医師の診療を受けることは、どう思いますか。

□利用してみたい
□利用したくない→その理由をお聞かせください
□体験したことがないから不安だから
□説明の声が聞き取りにくいから
□会った方が話しやすいから
□その他（　　　　　　　　　　　）

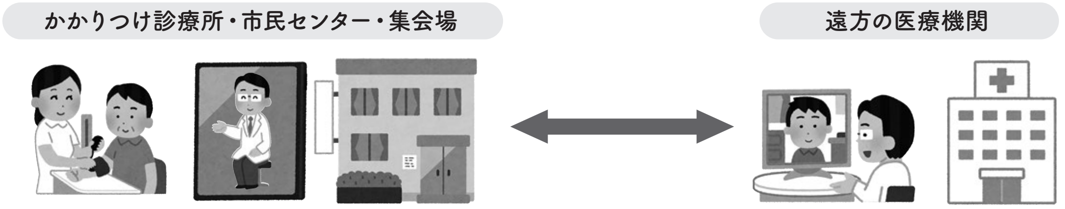

[問13] 自宅で、看護師や介護士が隣にいながら、テレビ電話で、かかりつけの医師の診療を受けることは、どう思いますか。

□利用してみたい
□利用したくない→その理由をお聞かせください
□体験したことがないから不安だから
□説明の声が聞き取りにくいから
□会った方が話しやすいから
□その他（　　　　　　　　　　　）

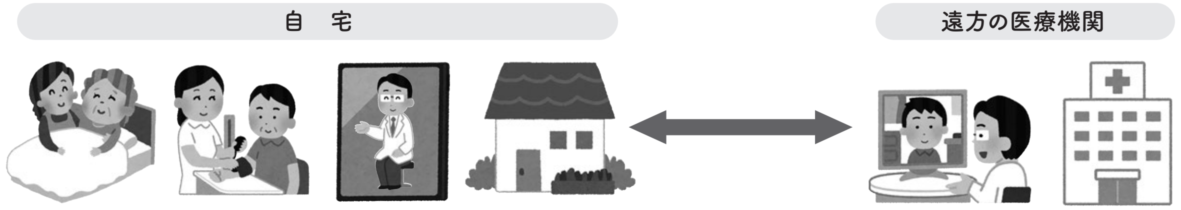

[問14] かかりつけの診療所や市民センター・集会所で、看護師が隣にいながら、テレビ電話で、薬剤師から薬の説明を受け、後日、薬が配送されたらどうですか？

□利用してみたい
□利用したくない→その理由をお聞かせください
□体験したことがないから不安だから
□説明の声が聞き取りにくいから
□会った方が話しやすいから
□その他（　　　　　　　　　　　）

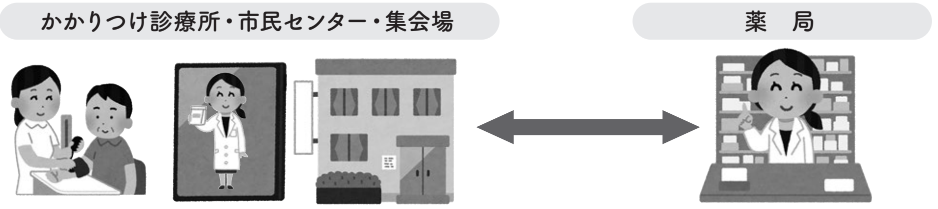

裏面もご覧ください
