# Supplementary material for: Telemedicine and Willingness to Die at Home in Rural and Remote Areas in Japan
Source: Telemed Rep. 2025 Sep 24;6(1):309–16. doi: 10.1177/26924366251382752 (PMC12547402; doi:10.1177/26924366251382752)
Supplement: Supplementary Table S1 [file 26924366251382752_supplementary_table_s1.pdf]

Supplementary Table 1.

Association between specific healthcare services needed for end-of-life care at home and willingness to die at home, adjusted for other covariates (N = 1,451)

Available online

**Variables**	**Adjusted odds ratio**	**p value** [a]
**Specific healthcare services**		
Telemedicine	1.41 (1.01–1.98)	0.045
Home doctor visits	1.50 (1.19–1.90)	<0.001
Home nursing care	1.26 (1.00–1.60)	0.053
Home personal care services	1.00 (0.76–1.30)	0.975
Home support services	0.81 (0.61–1.06)	0.117
Senior day care	0.97 (0.77–1.22)	0.796
**Covariates** (other factors)		
Sex (male)	1.89 (1.51–2.38)	<0.001
Age (20–59 years)	1.39 (1.02–1.89)	0.039
Residence area (remote island)	0.46 (0.26–0.82)	0.008
Household composition (living alone)	0.80 (0.59–1.08)	0.142
Mobile phone possession (smartphone)	0.68 (0.53–0.88)	0.003
Current medical consultation status (regular outpatient visits)	0.91 (0.70–1.19)	0.488
Advance care planning (experienced)	0.97 (0.77–1.22)	0.800

[a] Adjusted for all variables listed in the table.
